# Exploring the configuration spaces of surface materials using time-dependent diffraction patterns and unsupervised learning

**DOI:** 10.1038/s41598-020-62782-6

**Published:** 2020-04-03

**Authors:** Daniel M. Packwood

**Affiliations:** Institute for Integrated Cell-Material Sciences (iCeMS), Kyoto University, Yoshida-Honmachi, Sakyo-ku Kyoto, 606-8501 Japan

**Keywords:** Materials science, Mathematics and computing

## Abstract

Computational methods for exploring the atomic configuration spaces of surface materials will lead to breakthroughs in nanotechnology and beyond. In order to develop such methods, especially ones utilizing machine learning approaches, descriptors which encode the structural features of the candidate configurations are required. In this paper, we propose the use of time-dependent electron diffraction simulations to create descriptors for the configurations of surface materials. Our proposal utilizes the fact that the sub-femtosecond time-dependence of electron diffraction patterns are highly sensitive to the arrangement of atoms in the surface region of the material, allowing one to distinguish configurations which possess identical symmetry but differ in the locations of the atoms in the unit cell. We demonstrate the effectiveness of this approach by considering the simple cases of copper(111) and an organic self-assembled monolayer system, and use it to search for metastable configurations of these materials.

## Introduction

Innovative methods for exploring configuration spaces will enable structure predictions for materials from first-principles theory^1^. A configuration space, which describes the various spatial arrangements (configurations) that the atoms inside of a material might have, has dimensionality in the order of 3 *N*, where *N* is the number of atoms in the system. For all but the most trivial materials, the atomic configuration space is therefore high-dimensional, which makes the task of finding the ground state configuration and other thermodynamically accessible configurations extremely difficult without the use of judicious search strategies.

Recently, several interesting search strategies based on machine learning and genetic algorithms have been proposed to search the configuration spaces of atomic and molecular crystals^2–8^. In contrast to bulk crystals, relatively little attention has been paid to the cases of material surfaces or surface-adsorbed monolayers. While novel supervised learning^9–15^ and Monte Carlo strategies^16,17^ have been developed to search the configuration spaces of some special cases, efficient structure prediction is still far from being routine for these kinds of systems. This situation is unsatisfactory, because many forefront topics in experimental materials science, including nanotechnology^18^, thin-film electronics^19^, spintronics^20^, and even regenerative medicine^21^, rely on our ability to predict and manipulate the atomic structures of surfaces and surface monolayers.

Machine learning approaches to configuration space searching require descriptors (or, more directly, dissimilarity metrics) which compactly encode the structural features of the configurations. For the cases of bulk crystals or material surfaces, the choice of descriptor involves two difficulties. The first difficulty is that a majority of descriptors in the literature attempt to incorporate the relationship between atomic configuration and energy, typically through terms which have an inverse dependence on interatomic distances^22^. This inverse dependence is undesirable for configuration space searching, which inherently involves non-equilibrium configurations with very small inter-atomic distances. The inverse dependence causes such descriptors to explode in magnitude for strongly non-equilibrium configurations, which effectively creates outliers in the sample data and makes it difficult to extract trends via machine learning techniques. The second difficulty arises due to the lack of effective descriptors for periodic systems. While many prominent descriptors in the literature are based upon atomic positions or atomic fragments, these do not extend naturally to periodic systems and symmetry needs to be imposed *ad hoc* to prevent them from becoming infinite dimensional.

In this paper, we demonstrate how time-dependent electron diffraction patterns can be used as descriptors for the atomic configurations of material surfaces and organic monolayers. This approach is inspired by low-energy electron diffraction (LEED) experiments, in which the diffraction pattern of an electron beam from a surface provides a fingerprint for the surface’s atomic configuration. This ‘time-dependence’, which refers to the sub-femtosecond evolution of the diffraction pattern as the electrons pass through the detector, cannot be detected experimentally but can be simulated by integrating the time-dependent Schrodinger equation. By incorporating this time-dependence, we obtain descriptors with a strong sensitivity to the arrangement of atoms at the surface. Our approach contrasts with descriptors based upon time-averaged diffraction patterns, which are sensitive to the symmetry of the atomic configuration but do not strongly distinguish different configurations which have the same symmetry^23^. A challenging aspect of using time-dependent electron diffraction patterns for configuration space searching is that they are incompatible with machine learning methods which directly learn the relationship between atomic configuration and energy. This is because diffraction patterns do not incorporate any information related to energy. To overcome this problem we therefore adopt an unsupervised learning approach, in which time-dependent electron diffraction patterns are used to learn the main ‘structural features’ that are present in the configuration space. Optimization techniques are then used to predict metastable configurations with these structural features. By demonstrating that time-dependent electron diffraction patterns have sufficient sensitivity for configuration space searching, our work provides a much needed direction for developing machine learning approaches to surface structure prediction. This research direction also appears novel, bringing quantum dynamics into the exciting interaction that currently exists between data science and density functional theory.

## Results

### Time-dependent electron diffraction simulations

In a low-energy electron diffraction (LEED) experiment, an electron gun pointed at a crystalline surface ejects a beam of electrons with kinetic energies between 10–300 eV. Due to these low kinetic energies, the electrons quickly decelerate after reaching the surface region and do not penetrate far beyond the first few atomic layers. Furthermore, due to their wave-like nature, a small fraction of electrons diffract from the atoms near the surface and scatter back towards the electron gun. A detector placed behind the electron gun then records the electron diffraction pattern.

**Figure 1:**
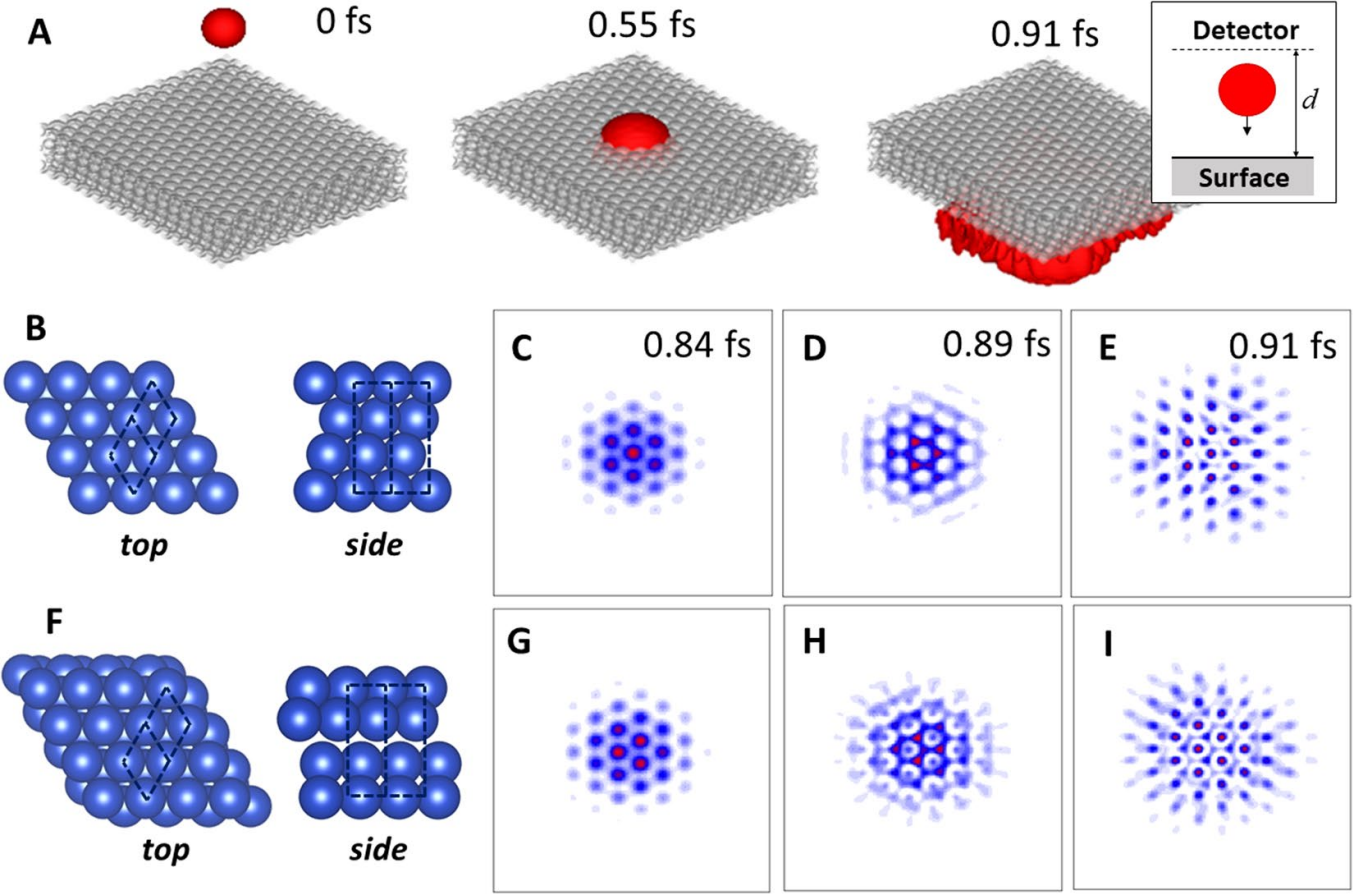
Simulation of time-dependent electron diffraction from a copper(111) (Cu(111)) surface. (**A**) Time evolution of a Gaussian wave packet (red). The red surface encloses 95% of the square amplitude of the wave packet. The grey surface encloses the region where the electrostatic potential is within 5% of its minimum value. Images created using the rgl package version 0.100.19^38^ and misc3D package version 0.8–4^39^. (**B**) Atom configuration of a four-layer Cu(111) surface. Blue spheres are Cu atoms and dotted lines indicate unit cells. Images created using VESTA version 3.4.4^43^ (**C–E**) Electron diffraction pattern for Cu(111) at various times. Blue and red indicate regions of low and high intensity, respectively. (**F**) Random configuration of Cu atoms with the same symmetry as Cu(111). (**G–I**) Electron diffraction pattern for the random configuration at the same times as C–E.

If observed on a fine enough time scale and with a very sensitive detector, the electron diffraction pattern would be time-dependent. Such time-dependence would arise as individual electrons scatter from the surface at different times, causing fluctuations in the peak intensities. Of course, such time-dependence cannot be observed in experimental settings, in which detectors of low time-resolution (typically micro- to milliseconds) are used and a time-averaged diffraction pattern is recorded by necessity. However, such restrictions do not apply in computational settings. Figure 1A shows a simulation of a 60 eV Gaussian wave packet penetrating through a copper(111) (Cu(111)) surface (of dimensions 34.42 Å by 40 Å). The atomic structure of Cu(111) is show in Fig. 1B. These simulations were performed by directly integrating the time-dependent Schrodinger equation using the finite-difference time-domain (FDTD) technique^24,25^. In these simulations, a time-independent electrostatic potential calculated from density functional theory (DFT) was used to model the wave packet-surface interaction (see methods). The surface is therefore modelled correctly (within the generalized gradient approximation), however its electron distribution does not change in response to the incoming wave packet. The latter approximation is widely used in simulations of low-energy electron diffraction (see 26,27). The simulations show that a majority of the wave packet (over 95% of its square amplitude) passes through the surface. Figure 1C–E show the wave packet amplitude present at the ‘detector’ as a function of time since the start of the simulation. In these simulations, the ‘detector’ is a 2D plane lying parallel to the surface and placed *d* = 9.07 Å above it. These figures confirm that a small amount of the wave packet undergoes diffraction and scatters away from the surface. The six-fold diffraction pattern expected of Cu(111) can be seen in these figures. Moreover, a rich, sub-femtosecond time-dependence in both the peak intensities and shapes can be observed.

Figure 1F shows the structure of a ‘randomized’ copper surface. This copper surface has the same symmetry (same unit cell shape and size) and same number of atoms per unit cell as ordinary Cu(111), however the positions of the Cu atoms have been chosen randomly, subject to some minor conditions (see methods). Figure 1G–I show the time-evolution of the electron diffraction pattern (wave packet square amplitude) observed at the detector. The same peaks as seen for the ordinary Cu(111) case in Fig. 1C–E appear, which is expected due to the identical symmetry of the two structures. However, the peak intensities and shapes for the randomized copper surface differ substantially from the ordinary Cu(111) case. This observation suggests that these time-dependent patterns contain rich information on the atomic configuration of the surface, rather than on surface symmetry alone, and could be used as descriptors for exploring the configuration space of surface materials.

### Dissimilarity metric and unsupervised configuration space searching for copper(111)

We now consider the use of time-dependent electron diffraction patterns for configuration space searching. A descriptor for any atomic configuration can be defined as the sequence of diffraction patterns observed from time *t* = *t*_0_ until time *t* = *t*_1_. In practical machine learning calculations, we need a measure of dissimilarity between descriptors. Letting *k* and *j* denote two atomic configurations, and *q*_*k*_(*x*, *y*, *t*) denote the wave packet density (amplitude per unit area) at point (*x*, *y*) on the detector at time *t* for configuration *k*, we define

1 $${d}_{kj}={\int }_{{t}_{0}}^{{t}_{1}}{({\int }_{D}{({q}_{k}(x,y,t)-{q}_{j}(x,y,t))}^{2}dxdy)}^{1/2}dt$$

as the dissimilarity between the descriptors of configurations *k* and *j*. In this definition, the inner integral over *D* is performed over the area of the detector. The dissimilarity metric in Eq. (1) satisfies all mathematical requirements of a distance metric (see methods). In this formalism the two times *t*_0_ and *t*_1_ can be set freely by the user. Ideally, we would set *t*_0_ = 0 and *t*_1_ = ∞, however due to shortcomings in the electron diffraction simulation it is preferable not to do this. This point will be discussed in later sections.

**Figure 2:**
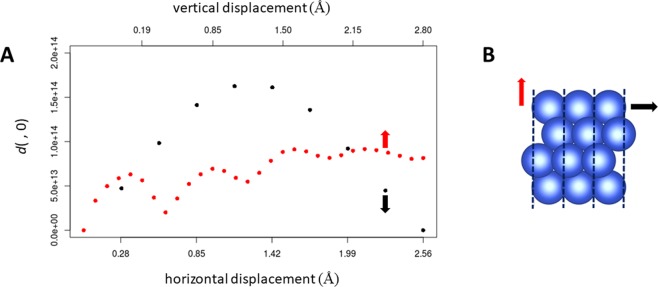
Variation of the dissimilarity metric (Eq. (1)) with respect to the true Cu(111) configuration. (**A**) Variation as the top atomic layer is shifted in the horizontal direction (black points) and as the top atomic layer is shifted in the vertical direction (red points). *d*(, 0) means that the dissimilarity metric relative to the true Cu(111) configuration. (**B**) True Cu(111) structure viewed from the side. Black and red arrows indicate the directions of the horizontal and vertical shifts, respectively, and dotted lines indicate unit cells. Image created using VESTA version 3.4.4^43^.

In order to confirm the sensitivity of these descriptors, we consider how Eq. (1) responds to shifts in the top plane of atoms in pristine Cu(111). Figure 2 plots Eq. (1) as a function of in-plane and out-of-plane shifts of the top layer of atoms in Cu(111). It can be seen that Eq. (1) varies in a parabolic manner with in-plane sliding, with the peak occurring at exactly half of a Cu(111) lattice vector. This is expected due to the symmetry of the Cu(111) surface. The dissimilarity metric is somewhat less sensitive to out-of-plane shifts, and exhibits an oscillatory variation. This variation is probably due to phase interference effects between the waves scattered from the top and second-top atomic layers of copper. This oscillatory variation means that the dissimilarity metric will have a reduced effectiveness for cases whose out-of-plane displacement differs by a period of this oscillation. Descriptors based upon time-dependent electron diffraction patterns are therefore strongly sensitive to horizontal shifts in the top layer of atoms, and somewhat less sensitive to vertical shifts, particularly when the vertical shift is comparable to a period of the oscillation shown in Fig. 2.

Having confirmed the sensitivity of these descriptors, we now evaluate their usefulness for configuration space searching. We do this in the following two steps. In the first step, we apply an unsupervised learning technique (hierarchical clustering), in which Eq. (1) is used to learn sets of ‘structural features’ which are present in the configuration space. In the second step, we use energy minimization techniques to obtain metastable configurations containing these structural features. In this context, ‘structural features’ broadly includes features arising from both the positions of the atoms and the electron distribution in the surface region, because electron diffraction patterns are determined by both of these together. This two-step strategy is preferred to trying to learn the mapping between atomic configuration and energy directly in a single step (as is done in supervised learning techniques, such as Gaussian regression^28^), because electron diffraction patterns do not contain any direct connection with energy.

**Figure 3:**
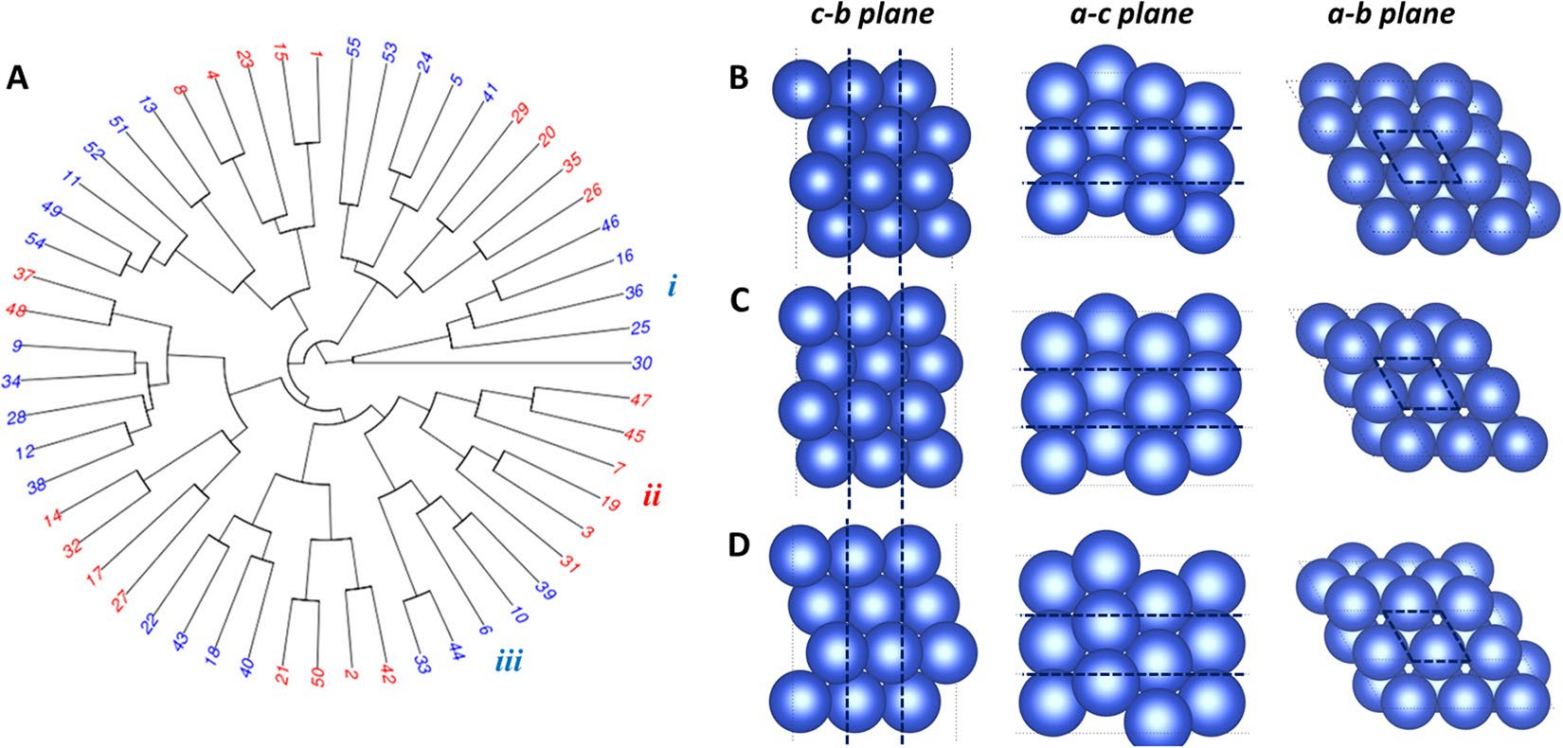
Searching for metastable configurations of Cu(111). (**A**) Circular dendrogram computed for 55 randomised copper configurations, each having the same symmetry as Cu(111), using the dissimilarity metric in Eq. (1). Alternating red and blue colors identify the clusters. (**B–D**) Metastable configurations of Cu(111) obtained by structure optimization within the clusters labelled *i*, *ii*, and *iii*, respectively (see text for details). The dotted back lines indicate the unit cell. Images created using VESTA version 3.4.4^43^.

In order to proceed with the first step (unsupervised learning), a sample of over 50 ‘randomized’ configurations of copper (111) were generated and the dissimilarity metric between each pair of them calculated. As before, these randomized configurations possessed the same symmetry and same number of atoms per unit cell as pristine Cu(111), but differ only in the locations of the atoms. Figure 3A shows a circular dendrogram computed using this dissimilarity metric and the hierarchical clustering method (see methods). The dendrogram arranges the copper configurations into so-called clusters, where configurations belonging to the same cluster are close together with respect to the dissimilarity metric. Twelve clusters can be identified from the whole diagram, as indicated by the alternating red and blue colors (also see Supporting Figure S1 and Table S1). Providing that Eq. (1) is an effective dissimilarity metric, each cluster should represent a distinct set of structural features that are present in the configuration space and learned from the sample data. In principle, the structural features represented by a cluster could be determined visually by examining each configuration in the cluster and noting their similarities. In the present case, however, the similarities and differences between the randomized configurations are difficult to detect by eye, suggesting that the ‘structural features’ learned by this method are relatively subtle.

For the second step (optimization), we attempt to predict metastable configurations which possess the structural features represented by the clusters. Let *C* denote a specific cluster (e.g., for cluster *i* in Fig. 3A, *C* = {46, 16, 36, 25, 30}). To identify a metastable configuration which possesses the structural features represented by *C*, we perform the interpolation

2 $${\bf{S}}=\sum _{k\in C}{c}_{k}{{\bf{S}}}_{k},$$

where the coefficients *c*_*k*_ are positive and sum to 1, and **S**_*k*_ denotes the *n* × 3 coordinate matrix of configuration *k*, where *n* is the number of atoms. Then, we optimize the coefficients in such a way that the total force acting on all atoms is minimized. The resulting configuration is one which possesses the structural features of that cluster, and is close to an energy minimum. We use Bayesian optimization to perform this optimization^28^, although other optimization methods could be used (see methods). To drive this configuration into the energy minimum, a local structure relaxation using DFT is then performed. The resulting metastable configuration should show similar structural features to the configurations in the original cluster, however differences may arise during the local structure relaxation. Note that this step only requires that 12 local structure optimizations (one for each cluster) be performed in order to carry out the configuration space search, compared to the 55 optimizations required if each of the configurations in the original sample were relaxed one-by-one (as in a brute-force search). However, the overall computational speed-up of the entire method is bottlenecked by the computational load of the diffraction simulations and the implementation of the Bayesian optimization. We will return to this point in the Discussion.

Figure 3B–D shows the metastable configurations found by this method from the clusters labelled as *i*, *ii*, and *iii*. These three metastable configurations are structurally distinct from one another, which can be confirmed by comparing their energies (−17.093 eV, −17.02 eV, and −17.153 eV, respectively) and inter-atomic angles (see Supporting Table S1). In fact, the metastable configuration obtained from cluster *iii* is quite similar to the true Cu(111) structure, having nearly the same energy and similar inter-atomic atomic angles (see Supporting Table S1). However, the second atomic layer from the surface for the structure obtained from cluster *iii* is slightly misplaced, causing discrepancies between some inter-atomic angles compared to true Cu(111). Interestingly, application of the above method to all clusters from Fig. 3A yields metastable configurations with energies close to either −17.093 eV, −17.02 eV, or −17.153 eV, but with clear structural variations between metastable configurations with similar energies (Supporting Table S1). This shows that the energy landscape for Cu(111), within the approximations of the DFT method used here (see the methods section), contains multiple metastable configurations, each possessing distinct structural features but residing at roughly only three distinct energy levels. The ability of this two-step approach to determine distinctive metastable configurations confirms that time-dependent electron diffraction patterns are useful for configuration space searching.

The prediction of the various metastable configurations above suggest that our descriptors are effective at distinguishing between configurations showing different structural features. However, it is also important to confirm that our descriptors are consistent between configurations which have similar structural features. An ideal way to confirm this consistency is to generate a ‘test’ atomic configuration which possesses the same structural features as one of the clusters, and then check whether it is placed into that cluster by the unsupervised learning procedure. Unfortunately, the subtle and elusive nature of the structural features determined above means that such atomic configurations cannot be selectively generated. We therefore used two alternative methods to confirm the consistency of our descriptors. In the first method, we computed the dendrogram in Fig. 3A again, but this time included the pristine Cu(111) structure as a ‘test’ configuration in the analysis as well. The new dendrogram is almost identical to the one shown in Fig. 3A, except that pristine Cu(111) structure is now included in the cluster *iii* (Supporting Figure S2). This result suggests that our descriptors are indeed consistent, because the metastable configuration yielded from the cluster *iii* had the closest energy, and presumably the most similar structural features, to pristine Cu(111).

In the second method, we use the metastable configurations predicted above as ‘test’ configurations, and determine where they are placed in the dendrogram during the unsupervised learning process. Providing that these configurations did not undergo extensive structural changes during the final local structure relaxation step (after optimization of Eq. (2)), they should retain the structural features of their original clusters and hence appear nearby or within these clusters during the unsupervised learning process. After performing these calculations, we find that 6 out of the 12 metastable configurations are placed into their original clusters, and that a further 4 are placed in positions nearby their original clusters (see Supporting Figure S3). Moreover, we find that the metastable configurations that were placed into their original clusters underwent less change during the local structure relaxation compared to those that were not placed into their original clusters (see Supporting Table S2). More specially, the root mean displacement of the atoms during the local relaxation, averaged over the 6 metastable states that were placed into their original clusters, was 0.41 Å ± 0.14 Å (error bounds are one standard deviation from the mean). This compares to a root mean square displacement of 0.58 Å ± 0.16 Å averaged over the states which were not placed into their original clusters. This is a sensible result, because metastable states which underwent less changes during the local relaxation should retain the structural features of their original clusters, and hence should appear in these clusters during the analysis. As well as validating the clustering shown in Fig. 3, this result provides strong evidence that our descriptors are indeed consistent between similar atomic configurations, at least for the case of Cu(111).

### Configuration searching for a self-assembled monolayer

Having demonstrated the use of our descriptors for the case of the clean material surface Cu(111), we now consider the case of the organic monolayer HO(CH_2_)_6_S ( = C_6_-SAM) covalently bonded to Au(111) *via* the S atom. Note that real LEED experiments on monolayers such as C_6_-SAM are not yet routine, probably due to the difficulty of preparing such systems under ultra-high vacuum as well as the instability of organic molecules to electron beams.

**Figure 4:**
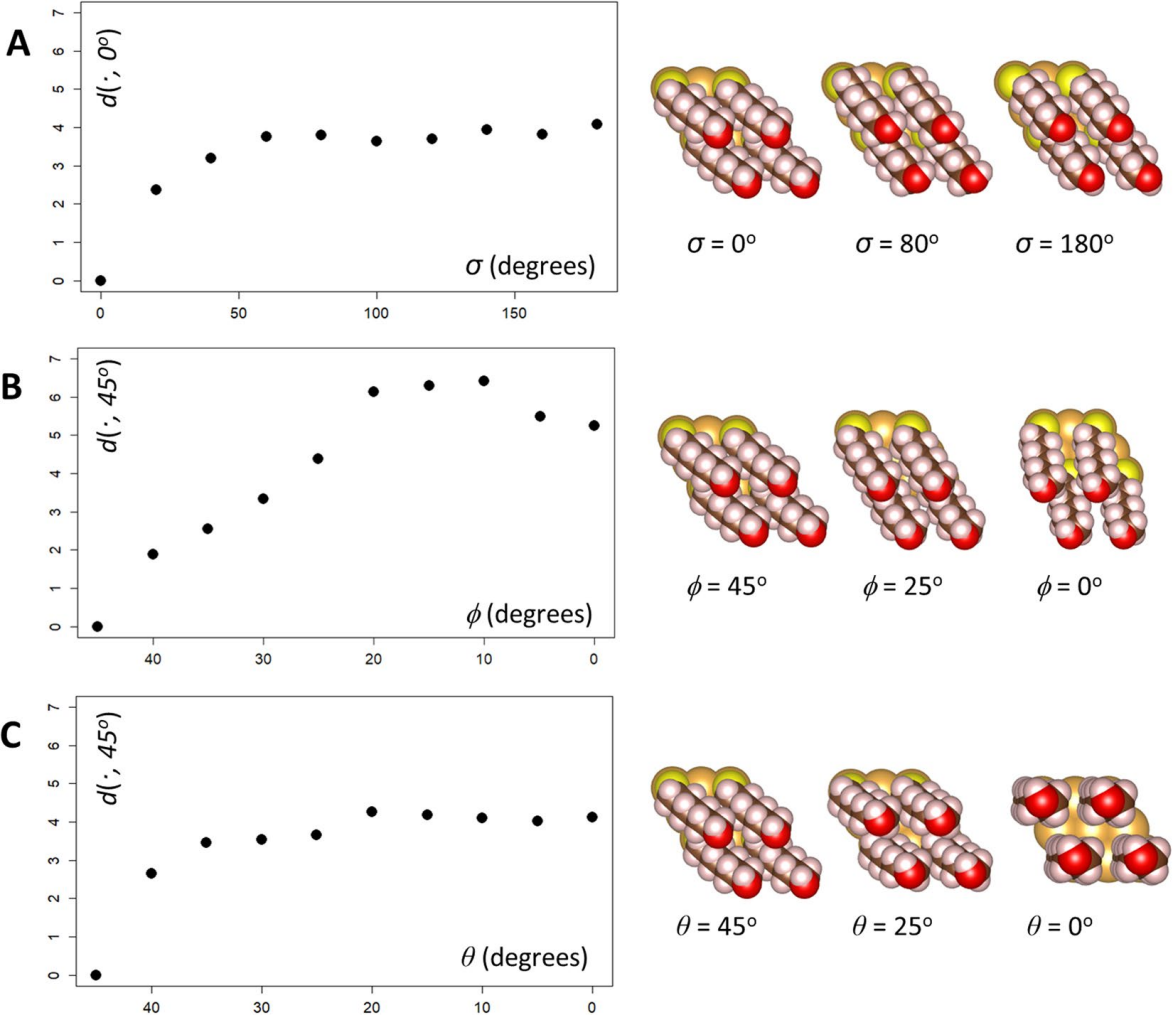
Variation of the dissimilarity metric with respect to the C_6_-SAM orientation. Gold spheres = gold atoms, yellow spheres = Sulfur atoms, white spheres = hydrogen atoms, brown spheres = carbon atoms, red atoms = oxygen atoms. (**A**) Variation with respect to the internal orientation *σ* about the S-O atom axis (computed with respect to the *σ* = 0° case). (**B**) Variation with respect to the azimuthal orientation *φ* of the S-O axis (computed with respect to the *φ* = 45° case). (**C**) Variation with respect to the elevation *θ* of the S-O backbone (computed with respect to the *θ* = 45° case. Note that *θ* = 0° indicates full elevation of the SAM backbone. Images created using VESTA version 3.4.4^43^.

Any configuration of the C_6_-SAM system can be roughly described by three continuous variables: one variable (*σ*) describing the internal orientation of the C_6_-SAM about the S-O atom axis, and two variables (*ϕ*, *θ*) describing the orientation of the C_6_-SAM backbone relative to the Au surface plane. Figure 4A–C plots the variation in the dissimilarity metric in Eq. (1) with respect to changes in these variables. These calculations consider a 2 × 2 supercell of an unrelaxed Au(111) surface possessing a single adsorbed C_6_-SAM molecule, where the molecule is in its relaxed gas-phase geometry. It can be seen that the dissimilarity metric is sensitive to changes in all three variables, with strongest sensitivity to the azimuthal angle *ϕ* that the SAM backbone makes to the Au surface plane (Fig. 4B). All three plots show that the dissimilarity metric rises and eventually reaches a plateau, indicating that it is most sensitive to small changes in configuration and less sensitive to large changes in configuration. The plateau occurs slowly for the variables *σ* and *ϕ* (Fig. 4A,B; note that *σ* is plotted across a wider range of values than *ϕ* or *θ*) and most quickly for the variable *θ*, which corresponds to the elevation of the SAM backbone relative to the Au surface plane (Fig. 4C). Taken together, these results indicate that the dissimilarity metric is most sensitive to differences in internal orientation and azimuthal orientation of the SAM backbone, and relatively less sensitive to differences in the elevation of the SAM backbone. In each case, the variation of the dissimilarity metric exhibits small kinks and is not smooth. This may be due to charge transfer effects at the surface, which are known to vary in a non-linear manner as SAM orientation is changed^29^. No dramatic oscillations such as the ones seen for bare Cu(111) are observed for this system, suggesting that time-dependent electron diffraction patterns may be more effective for the present system.

**Figure 5:**
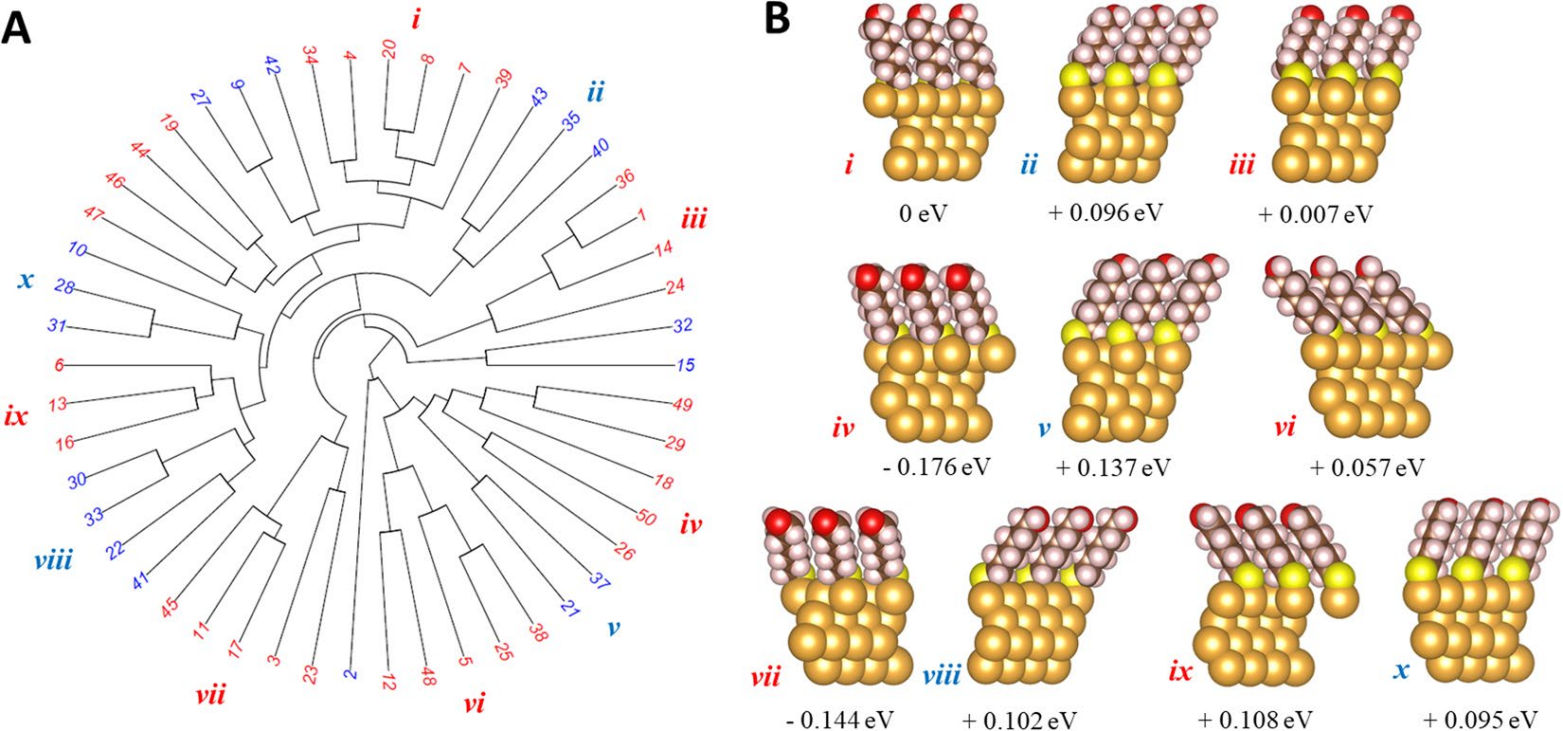
Exploring the configuration space of C_6_SAM monolayer on a gold(111) (Au(111)) surface. (**A**) Circular dendrogram computed for 50 Au(111)-C_6_SAM configurations, with the C_6_SAM orientation set randomly. Alternating red and blue colors identify the clusters. (**B**) Metastable configurations of the C_6_SAM monolayer obtained from optimization of the clusters in in (**A**). The configurations are shown as side-views. The numbers are the energy of each configuration with respect to configuration *i*. Images created using VESTA version 3.4.4^43^.

To illustrate how these descriptors might be used for configuration space searching, we again employ the two-step scheme from the previous section involving unsupervised learning and optimization. For the first step (unsupervised learning), we generated a sample of 50 different C_6_-SAM configurations, each with randomly chosen orientations. A visual inspection of the dendrogram suggests roughly 14 clusters, which are indicated by alternating colors in Fig. 4. As with Cu(111) above, each cluster should correspond to a set of distinct ‘structural features’ in the configuration space. For the second step (optimization), we again use Eq. (2) to search for metastable configurations containing structural features identified by the clusters (in this case, the quantities **S**_*k*_ in Eq. (2) are vectors consisting of the three variables (*σ, ϕ*, *θ*) described above). Metastable configurations obtained *via* this method from several clusters are displayed around the periphery of the dendrogram in Fig. 5B (metastable configurations from other clusters are shown in Supporting Table S3). These metastable configurations display a variety of internal orientations, azimuthal angles, and elevations, and no obvious similarities between metastable configurations can be found. The identification of distinct metastable configurations *via* this method again supports the use of time-dependent electron diffraction patterns for configuration space searching and structure predictions. As before, this step only requires that 14 local structure optimizations (one for each cluster) be performed compared to the 50 optimizations required if each of the configurations in the original sample were relaxed one-by-one.

Finally, we again show that our descriptors are consistent for this system by validating the clustering shown in Fig. 5A. As was done for the Cu(111) case, we use the metastable configurations predicted above as ‘test’ cases, and check whether or not they appear within or nearby their original cluster during the unsupervised learning process. We find that 7 out of the 14 metastable configurations are placed into their original clusters, and that 2 other metastable configurations are placed nearby their original clusters (Supporting Figure S4). Moreover, we again find that the metastable configurations which were placed into their original clusters underwent considerably less change during the local structure relaxation (Supporting Table S4). Concretely, we obtain an average root mean square displacement of 0.042 Å ± 0.011 Å during relaxation for those placed back into their original clusters, compared to 0.162 Å ± 0.058 Å for those that were not. Thus, metastable configurations which underwent less structural relaxation, and hence retained the structural features of their original cluster, were placed back into these clusters during the analysis as expected. These results again confirm that our descriptors show consistent behavior for atomic configurations possessing similar structural features.

## Discussion

The results presented above show that descriptors based upon time-dependent electron diffraction patterns are indeed sensitive to variations in atomic configuration, and supports their use for searching atomic configuration spaces. A difficulty with these descriptors is that they have no direct connection with energy, and therefore are incompatible with supervised learning techniques (such as kernel regression or Gaussian regression) which attempt to learn how atomic configurations map to energy. For configuration space searching, it is therefore more appropriate to take an unsupervised learning approach, in which sets of ‘structural features’ are learned from a data sample, and then used as a basis for predicting metastable configurations. While the specific technique used here (identification of sets of ‘structural features’ by use of unsupervised learning, followed by optimization of a linear combination structure to find a metastable configuration) does not have a specific name in the machine learning literature, it mimics the flow of supervised learning methods which search configuration spaces by learning the energy function directly (namely, identification of low-energy regions of the configuration space, followed by local searching within those regions). In particular, this technique satisfies one of the main conditions of efficient configuration space searching – minimization of the number of costly *ab initio* local structure relaxations – making it a realistic means to illustrate the application of time-dependent diffraction-based descriptors and dissimilarity metrics. With the exclusion of very small systems, such an approach is more efficient than performing an *ab initio* structure relaxation on every configuration in a large sample.

At present, there are two weaknesses of using time-dependent diffraction patterns as descriptors. The most obvious one is the long calculation times required to perform the electron diffraction simulations. A single electron diffraction simulation using multiple processors can require 1–3 hours of computational time, depending upon the mesh size and time step. However, the situation may not be hopeless: the method used to perform these calculations in this paper (the FDTD method) is only one of many methods developed to solve the time-dependent Schrodinger equation, and another method may result in a substantial reduction in computational time compared to the FDTD technique. Indeed, a new method for solving the time-dependent Schrodinger equation, especially one designed to maximize the accuracy of the simulated diffraction pattern rather than the entire wave packet, should result in dramatic computational savings. Before the electron diffraction simulations, an electrostatic potential must be computed from DFT. While these calculations can be performed quickly for the system sizes considered in this paper, they become demanding for the cases involving large unit cells and many atoms. One possibility to reduce this computational time is to perform non-self-consistent calculations using an electron density composed of a superposition of atomic orbital electron densities. In Supporting Figure S5, we compare dendrograms computed using self-consistent and non-self-consistent electrostatic potentials. For the case of Cu(111), the two calculations yield virtually identical dendrograms, with only minor differences in the values of the dissimilarities being seen. However, for the case of C_6_-SAM on Au(111), the two dendrograms show substantial differences. These results suggest that electrostatic potentials may be computed in a non-self-consistent manner for purely metallic systems, in which the charge transfer and bond formation is not significant and configurations can be compared on the basis of atom positions alone. However, for systems containing organic molecules, charge transfer and bond formation is substantial and should be considered when comparing configurations. Regardless of this fact, non-self-consistent approaches or other approximate first-principles methods are probably the only reasonable way to compute the electrostatic potential for large systems at sufficiently low computational cost. For this reason, it would be worthwhile to study the extent to which such approximations can be applied to organic monolayers and similar systems in future research.

The second weakness of our approach is due to an intrinsic limitation of electron diffraction simulations. Because these simulations are necessarily performed with a finite simulation cell, the incorporation of periodic boundary conditions or reflecting boundaries means that the electron wave packet eventually ends up interacting with itself. This self-interaction effect results in a complicated wave interference pattern at long simulation times, which obscures the true diffraction pattern from the atoms of the surface. In the present study, we dealt with this by introducing a complex absorbing potential (CAP) near the boundaries of the simulation box, which forces the electron wave function to decay near the boundary region^30,31^. While the incorporation of a CAP alleviates the self-interaction effect, it does not eliminate it entirely. Indeed, because the self-interaction problem is an entirely physical consequence of using a finite-sized simulation box, it seems doubtful that any mathematical trick could prevent it completely. For this reason, we recommend placing a finite upper limit *t*_1_ on the integral in Eq. (1).

Machine learning-based configuration space searching and structure predictions require consistent and sensitive descriptors for the atomic configurations. At present, such descriptors metrics are lacking for periodic systems, particularly material surfaces and surface monolayers. The advance of this paper is the concept that the sub-femtosecond time dynamics of electron diffraction patterns can be used to obtain consistent descriptors with strong sensitivity to variations in atomic configuration. To this end, we showed that descriptors constructed from time-dependent electron diffraction patterns can effectively distinguish between candidate atomic configurations for material surfaces and surface monolayers. Furthermore, we demonstrated how such descriptors can assist the search for metastable and ground state atomic configurations for these types of systems. While the computational demands for computing such descriptors are significant for systems involving large unit cells and numbers of atoms, these demands will be reduced with the development of effective numerical schemes for the time-dependent Schrodinger equation and approximate methods for computing electrostatic potentials. By inspiring such developments and encouraging further research on time-dependent diffraction patterns for machine learning applications, we hope that this work will lead to a new phase of materials science in which surface structure of real materials can be routinely predicted and controlled in experimental settings.

## Methods

### DFT calculations

All DFT calculations were performed with the Vienna Ab Initio Simulation Package (VASP)^32^. Electrostatic potential calculations and structural relaxations for the Cu(111) systems used four atomic-layer, single unit-cell slabs with 70 Å vacuum spaces, 550 eV basis set cut-offs, 4 × 4 × 1 Γ-centered *k*-points grids, and the PBE exchange-correlation functional^33^. All atom coordinates were translated so that the atoms in the top layer had vertical coordinate 28.35 Å.

Electrostatic potential calculations and structural relaxations for the Au-C_6_-SAM systems used four-layer, 2 unit cell x 2 unit cell Au(111) slabs with 70 Å vacuum spaces, 650 eV basis set cut-offs, 4 × 4 × 1 Γ-centered *k*-points grids, and the rev-vdW-DF2 exchange-correlation functional^34–36^. The Au slab was positioned so that one of the atoms in the top layer resided at the origin of the simulation box. The C_6_ molecule was then placed so that the S atom resided at exactly 2.3 Å above this gold atom. All atom coordinates were then shifted so that the top-most atom in the C_6_ molecule had vertical coordinate 28.35 Å.

For all systems, all atoms were allowed to move during the structural relaxations. Prior to electron diffraction simulations, the electrostatic potentials obtained above were expanded into 15 × 15 × 1 supercells for both the Cu(111) and the Au-C_6_-SAM systems. To save computer memory, the expanded electrostatic potentials were stored on sparse grids. Linear interpolation between the original grid point values as used to determine the value of the potential on the sparse grid. The number of grid points for the sparse grids was 240 × 240 × 420 for both the Cu(111) and Au-C_6_-SAM systems. This corresponded to simulation cells of dimension 35.42 Å × 40 Å × 70 Å and 79.91 Å × 92.28 Å × 70 Å, respectively.

### Electron diffraction simulations

A wave packet simulator using the finite-difference time-domain (FDTD) technique described in 24 was programmed in C++. These calculations were performed on the sparse grids obtained from the expanded electrostatic potentials described above. The initial wave packet had the form

3 $$\psi ({\bf{r}},t=0)=\frac{1}{\sqrt{2\pi {\sigma }^{2}}}{e}^{-\frac{{|{\bf{r}}-{{\bf{r}}}_{0}|}^{2}}{2{\sigma }^{2}}}{e}^{-2\pi i({r}_{z}-{r}_{z}^{0})/\lambda ({E}_{k})},$$

where the parameters **r**_0_, *σ*, and λ(*E*_*k*_) are the location of the center position, spread, and wavelength of the wave packet, respectively. *r*_*z*_ and *r*_*z*_° are the vertical components of **r** and **r**_0_, respectively. The wavelength is defined as

4 $$\lambda ({E}_{k})=\sqrt{2{\hslash }^{2}{\pi }^{2}/{m}_{e}{E}_{k}},$$

where *ℏ* is the reduced Planck constant, *m*_*e*_ is the electron mass, and *E*_*k*_ the kinetic energy of the wave packet, respectively. The initial wave packet is set by specifying the values of **r**_0_, *σ*, and *E*_*k*_. For the Cu(111) systems, these parameters were set such that **r**_0_ had vertical coordinate 42 Å and was projected onto the middle of the surface, *σ* = 1.77 Å, and *E*_*k*_ = 60 eV, respectively. For the Au-C_6_-SAM systems, these parameters were set such that **r**_0_ had vertical coordinate 42 Å and was projected onto the middle of the surface, *σ* = 7.99 Å, and *E*_*k*_ = 15 eV, respectively.

Before commencing wave packet propagation, a complex absorbing potential (CAP) region of size roughly 167 Å was added to the top and bottom of the simulation cell. In the CAP region, the electrostatic potential had a constant value (determined by its value at the top and bottom of the original simulation cell), and the CAP had the form

5 $$u({\bf{r}})=-\,i({e}_{c}\alpha /{h}_{z}){|{r}_{z}-{z}_{cut}|}^{2},$$

where *e*_*c*_ is the electron charge, *α* a constant, *h*_*z*_ the grid spacing in the vertical direction, *r*_*z*_ is the vertical component of **r**, and *z*_*cut*_ is the point along the vertical axis where the CAP region in question begins (i.e., *z*_*cut*_ was set to 0 for the bottom CAP region, and *z*_*cut*_ was set to the height of the original simulation cell for the top CAP region). The CAP Eq. (5) was set to 0 for all points outside of the CAP region. For all systems, we set *h*_*z*_ = 0.167 Å and *α* = 10^8^ eV/Å.

For all systems, wave packet propagation was performed for 8000 time steps. For the Cu(111) systems, the time steps had length 0.379 as. For the Au-C_6_-SAM systems, the time steps had length 0.482 as. The ‘detector’ for the electron diffraction was placed at vertical coordinate 37.42 Å for the Cu(111) systems and 45.44 Å for the Au-C_6_-SAM systems.

To reduce storage requirements, electron diffraction simulations only output the intensity at the detector at every 20^th^ time step.

#### Proof that *d*_*kj*_ is a metric

The term in the brackets is an *L*_2_ norm. Writing the time integral as the limit of a Riemann sum then shows that *d*_*kj*_ is a sum of *L*_2_ norms, and hence is an *L*_2_ norm itself.

#### Processing of results

The output of the electron diffraction simulations were processed using a script written in the R language^37^. Wave packets and electron diffraction patterns were plotted with the aid of the rgl^38^ and misc3D^39^ packages. Plotting and dissimilarity metric calculations were accelerated with the parallel computing packages foreach^40^ and doParallel^41^ for R.

When computing the dissimilarity metric in Eq. (1), we only integrated over the frames output from the electron diffraction simulation. We set *t*_0_ = 0.75 fs and *t*_1_ = 0.98 fs for all cases involving Cu(111) systems. For the Au-C_6_SAM systems discussed in Fig. 4 we used *t*_0_ = 1.03 fs and *t*_1_ = 2.40 fs. For the Au-C_6_SAM systems discussed in Fig. 5, we used *t*_0_ = 1.24 fs and *t*_1_ = 2.40 fs.

#### Automation of calculations

The results shown in Figs. 2–5 were obtained in an automated manner *via* an R script. This script looped over the following four steps: (1) Random generation of a Cu(111) structure, or random orientation of the C_6_ molecule on Au(111). (2) Submission of structure to VASP for electrostatic potential calculation. (3) Processing and expansion of the electrostatic potential. (4) Submission of expanded electrostatic potential to the wave packet simulator described above. In Step, (1) the random four-layer copper(111) configurations were constrained so that the distance between the top and bottom layers never exceeded 7.35 Å.

#### Bayesian optimization

Bayesian optimization of the coefficients (2) was performed using feature vectors comprised of the coefficients themselves, Gaussian kernels, and initial datasets consisting of ten random choices of coefficients and the energies of the corresponding configurations. The hyperparameters for the Gaussian kernels were set *via* marginal likelihood maximization using the initial dataset, and were not changed during successive iterations. Bayesian optimization ran for 50–100 iterations to find the optimal coefficients. The entire algorithm, using a new training set each time, was ran 5–100 times, and the best set of coefficients from all runs was used for subsequent calculations. This number of iterations and repetitions was excessive, because the optimal coefficients were typically found within 20–50 iterations of a single run.

#### Unsupervised learning

Hierarchical clustering was performed using the hclust function from R^37^ with complete linkage. The circular dendrograms were plotted using the ape package for R^42^.

## Supplementary information

Supplementary information.

## Data Availability

The datasets generated and analysed during the current study are available from the corresponding author on reasonable request.
