# Supplementary material for: Exploring the configuration spaces of surface materials using time-dependent diffraction patterns and unsupervised learning
Source: Sci Rep. 2020 Apr 3;10:5868. doi: 10.1038/s41598-020-62782-6 (PMC7125295; doi:10.1038/s41598-020-62782-6)
Supplement: Supplementary file 1 — Supplementary information. [file 41598_2020_62782_MOESM1_ESM.pdf]

Supporting information for:

Exploring the configuration spaces of surface materials using time-dependent diffraction patterns and unsupervised learning

Daniel M. Packwood

Institute for Integrated Cell-Material Sciences (iCeMS), Kyoto University,
Yoshida-Honmachi, Sakyo-ku, Kyoto, 606-8501, Japan

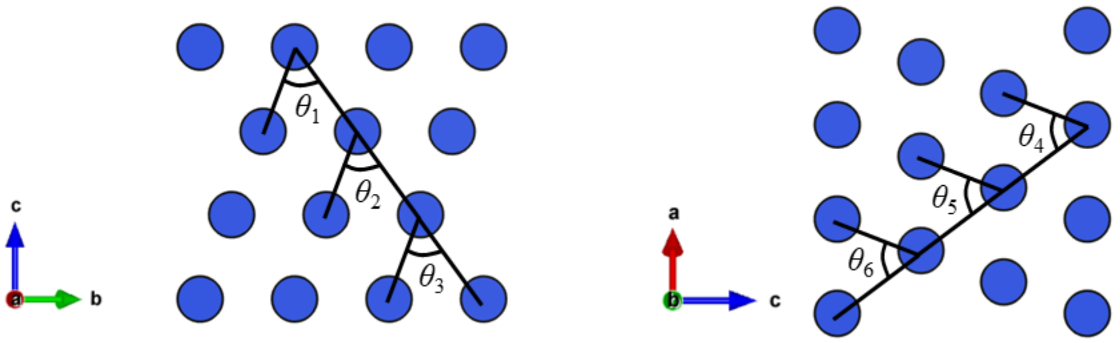

Figure S1. **Cu(111) structure**. 2 x 2 x 1 supercell of Cu(111) viewed along the a axis (left) and b axis (right). The angles shown above are defined for the three atoms closest to the viewer.

Cluster	*E* (eV)	θ_1 (º)	θ_2 (º)	θ_3 (º)	θ_4 (º)	θ_5 (º)	θ_6 (º)
46 – 30	-17.093	62.41	63.06	62.48	46.35	62.89	62.14
47 – 31	-17.020	62.35	62.83	62.57	62.64	63.03	62.59
39 – 33	-17.153	62.69	46.44	62.55	62.28	63.19	46.02
42 – 21	-17.020	45.45	46.48	46.20	62.87	62.56	62.73
40 – 22	-17.094	62.60	46.26	45.73	46.06	63.18	62.42
27 – 14	-17.096	46.31	63.05	62.34	46.32	63.08	62.56
38 – 9	-17.094	46.35	45.83	62.47	62.02	63.43	62.95
48 – 37	-17.095	62.84	46.34	46.31	45.82	62.93	62.07
54 – 13	-17.095	62.39	63.27	45.66	62.11	62.96	45.66
8 – 1	-17.152	46.56	63.01	62.47	62.80	46.75	62.66
55 – 41	-17.152	62.40	46.77	62.32	46.42	62.84	62.69
29 – 26	-17.094	46.25	63.02	62.28	62.45	46.54	62.56
True Cu(111)	-17.153	46.10	63.10	62.60	62.50	63.10	46.10

Table S1. **Optimised structures obtained from each of the clusters shown in Figure 3A of the main paper**. 'True Cu(111)' is the experimental Cu(111) configuration. *E* is the energy of the configuration (obtained from DFT), and the angles θ are defined in Figure S1. The blue, green, and red colors indicate structures with energies close to -17.02 eV, -17.094 eV, and -17.153 eV, respectively.

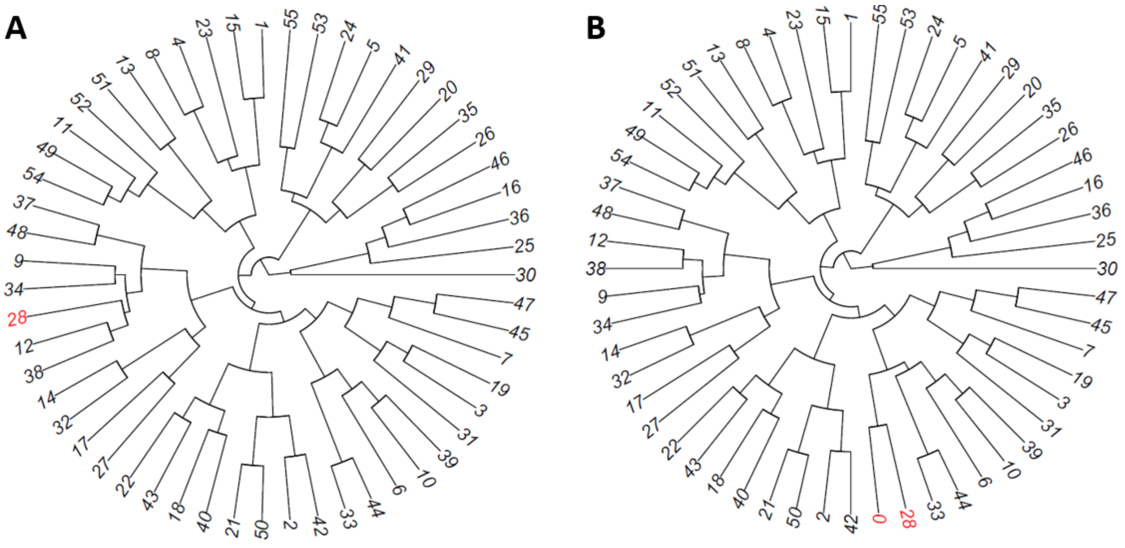

Figure S2. **u(111) dendrogram with real Cu(111) configuration included.** (A) The same dendrogram as in Figure 3A, (B) The dendrogram re-calculated with inclusion of the true Cu(111) configuration. The true Cu(111) structure is labelled as ‘0’ and drawn in red. Structure 28, also drawn in red, moves upon re-calculation of the dendrogram, however other structures remain in their original places.

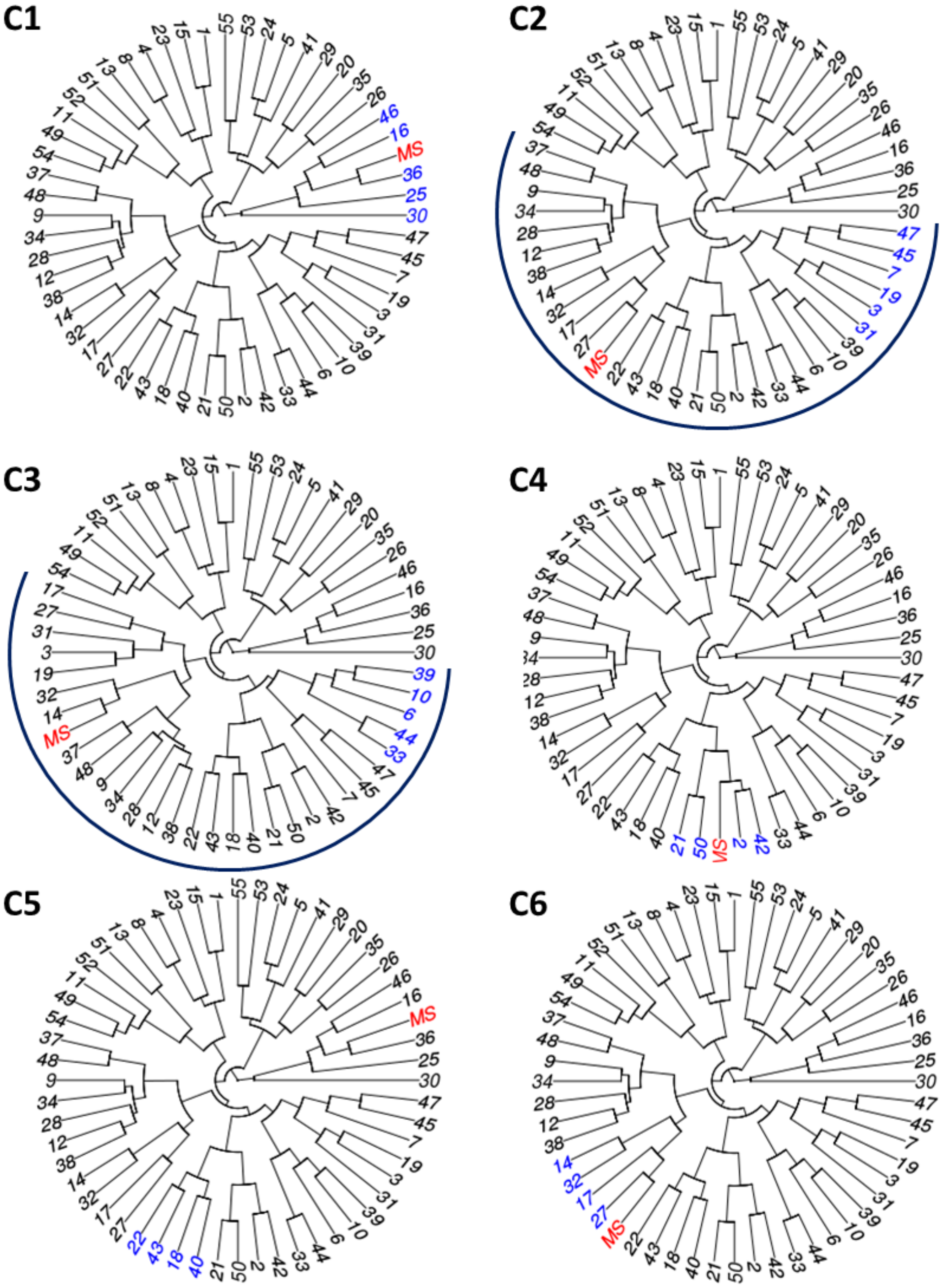
C1
C2
C3
C4
C5
C6

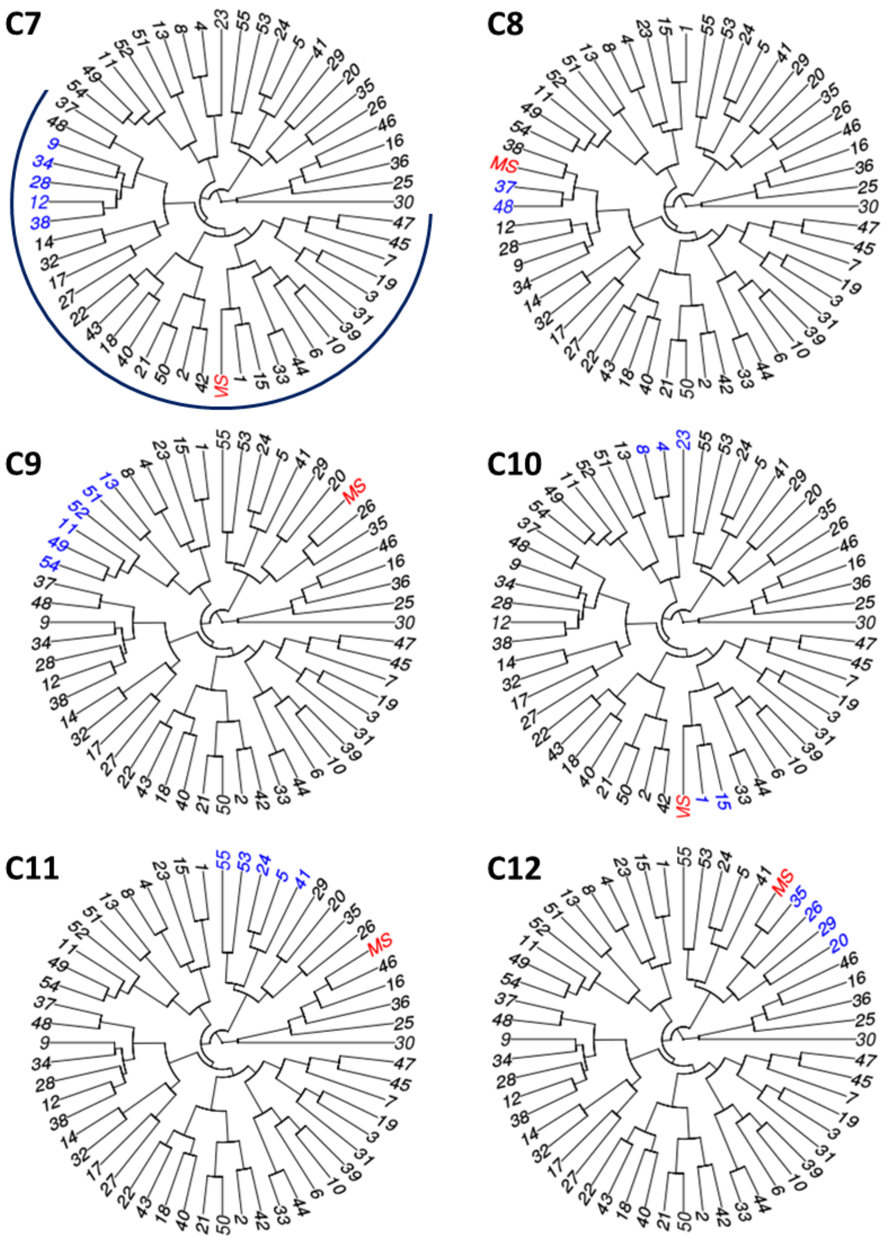

Figure S3. **Assignment of Cu(111) metastable states to clusters.** Same dendrogram as shown in Figure 3A, but computed with the inclusion of the metastable configurations obtained from each cluster. The original clusters are indicated by blue, and the metastable configuration indicated by the red label ‘MS’. The symbols C1, C2, …, C12 are labels for the clusters. The blue curves indicate the supercluster to which the cluster belongs to. Metastable configurations which are not placed within their original clusters but belong to the same supercluster can be considered as being nearby the original cluster. Note that the cluster C8 was broken up during the hierarchical clustering procedure after inclusion of the metastable configuration.

**Cluster number**	**RMSD (Å)**	**Classification**
C1	0.247	O
C2	0.315	X
C3	0.663	X
C4	0.393	O
C5	0.597	X
C6	0.355	O
C7	0.491	X
C8	0.358	O
C9	0.742	X
C10	0.481	O
C11	0.676	X
C12	0.648	O

Table S2. **Root mean square displacement of atoms during local structure relaxation to obtain the metastable configuration (Cu(111) configurations)**. C1, C2, …, C12 are cluster labels (see Figure S3). O and X indicate that the relaxed configurations were placed into the correct cluster and incorrect cluster, respectively, upon repeating the hierarchical clustering process, as shown in Figure S3.

Cluster	*E* (eV)	View along a-axis	View along c-axis
49 - 26	-134.799	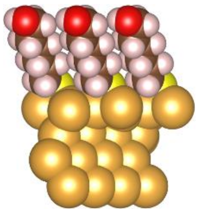	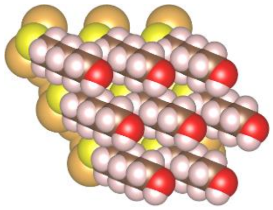
37, 21	-134.486	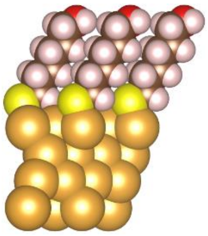	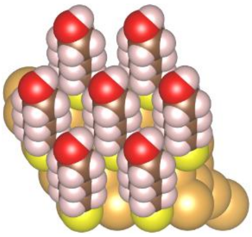
38 – 12	-134.566	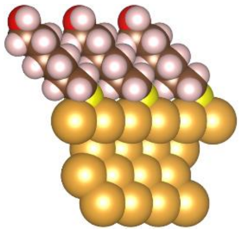	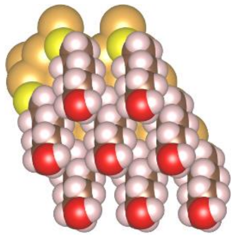
2	-134.497	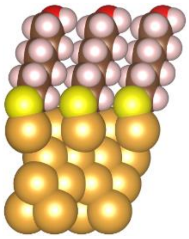	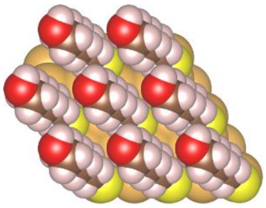
45 – 23	-134.767	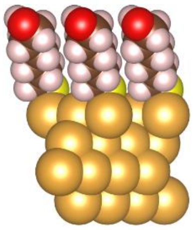	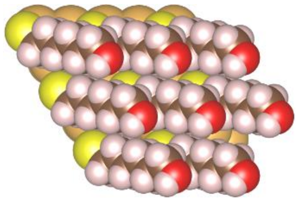
30 – 41	-134.521	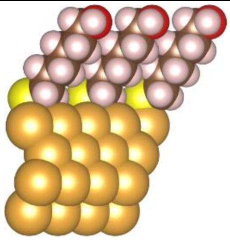	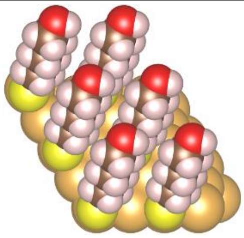

**Cluster**	***E*** **(eV)**	**View along a-axis**	**View along c-axis**
6 – 16	-134.514	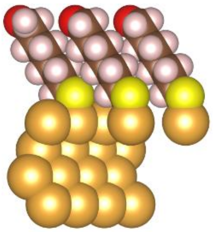	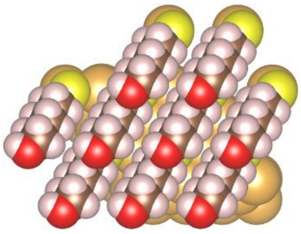
10 – 31	-134.528	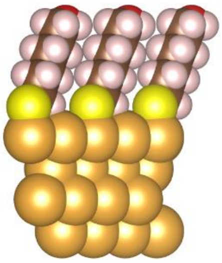	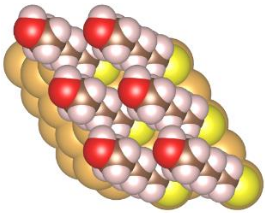
42 – 27	-134.783	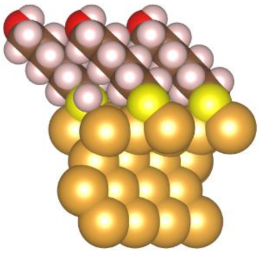	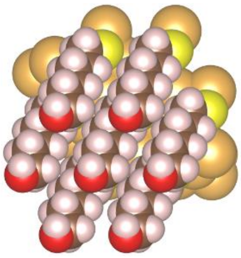
46 – 47	-134.618	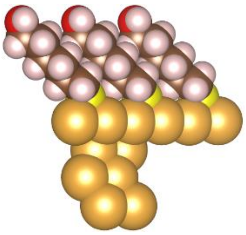	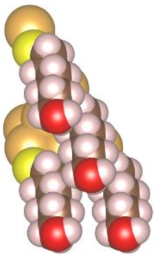
34 – 39	-134.623	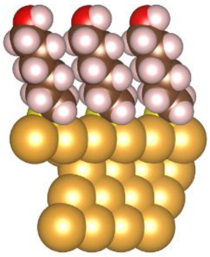	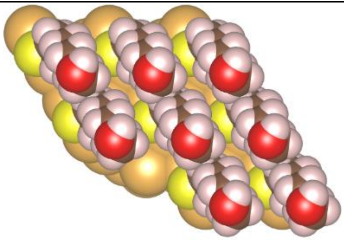
43 – 40	-134.527	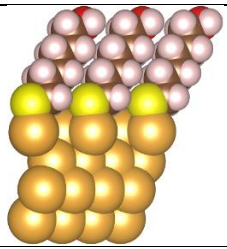	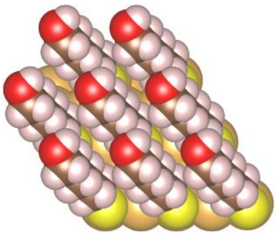

Cluster	*E* (eV)	View along a-axis	View along c-axis
36 – 24	-134.616	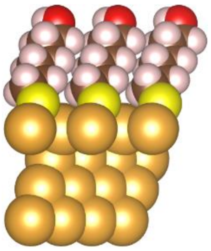	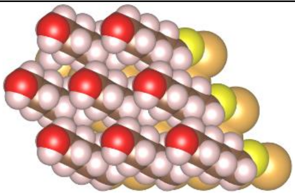
32, 15	-134.531	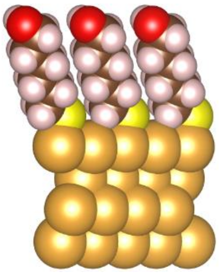	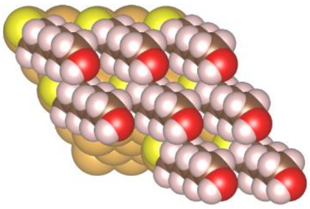

Table S3. **Optimised C_6-SAM structures obtained from each of the clusters shown in Figure 5A of the main paper**. *E* indicates the energy of each structure. The structures were drawn as 2 x 2 x 1 supercells. Images created using VESTA version 3.4.4 (30)

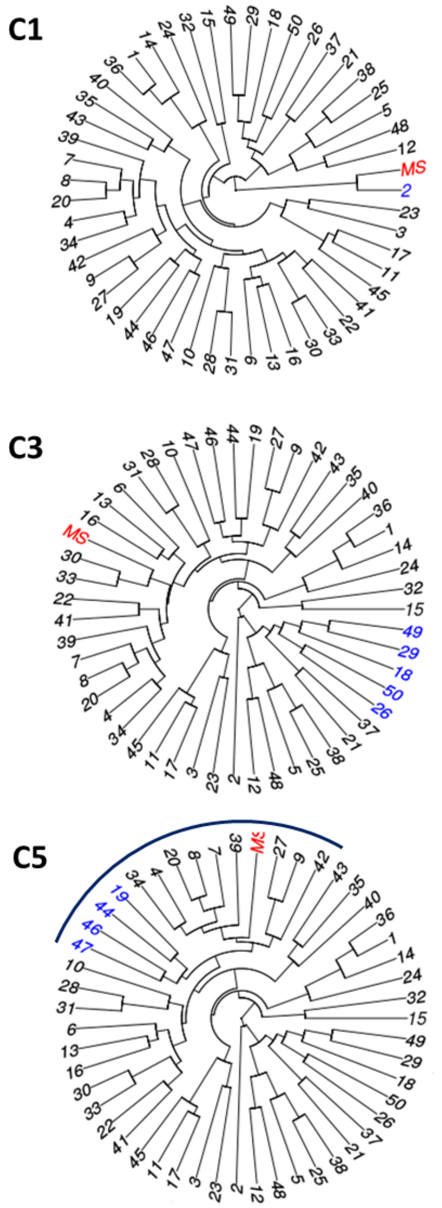
C1
C2
C3
C4
C5
C6

C7

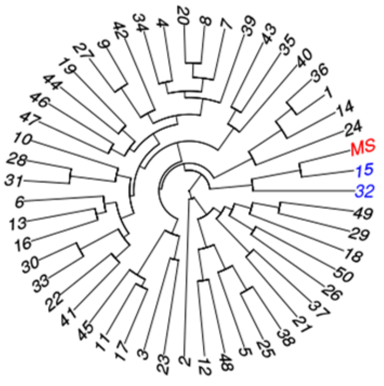

C8

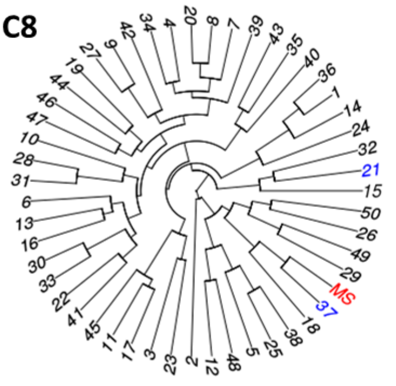

C9

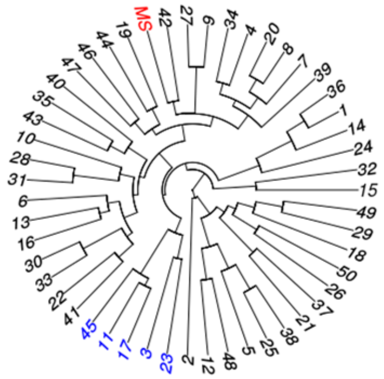

C10

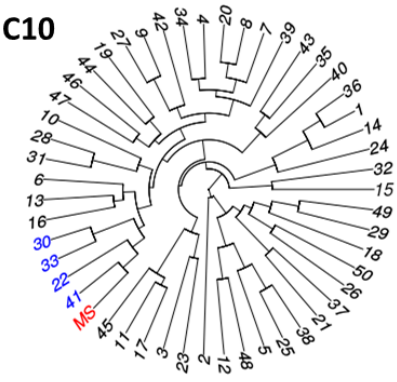

C11

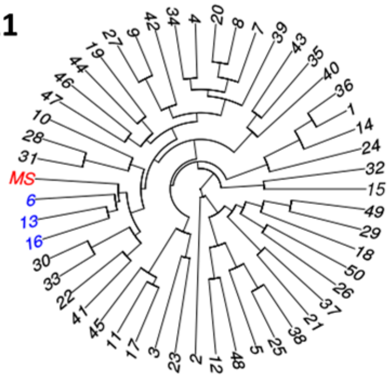

C12

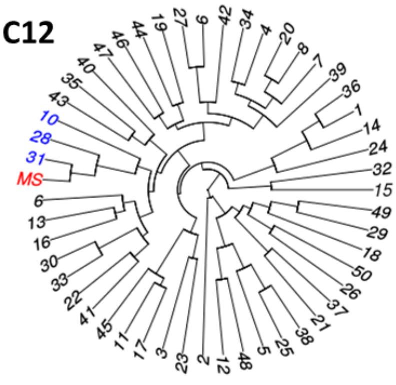

C13

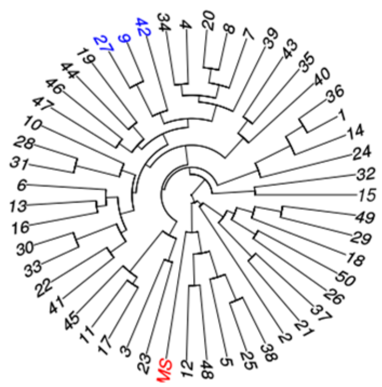

C14

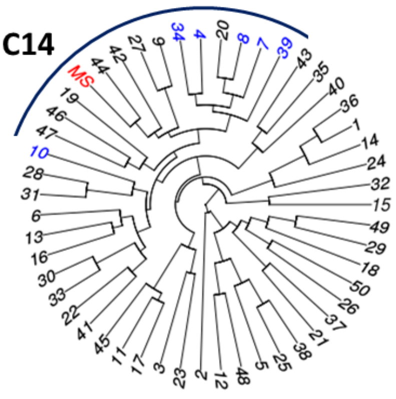

Figure S4. **Assignment of C_6-SAM metastable states to clusters.** Same dendrogram as shown in Figure 5A, but computed with the inclusion of the metastable configurations obtained from each cluster. The original clusters are indicated by blue, and the metastable configuration indicated by the red label 'MS'. The symbols C1, C2, …, C14 are labels for the clusters. The blue curves indicate the supercluster to which the cluster belongs to. Metastable configurations which are not placed within their original clusters but belong to the same supercluster can be considered as being nearby the original cluster. Note that the cluster C14 was broken up during the hierarchical clustering procedure after inclusion of the metastable configuration.

Cluster number	RMSD (Å)	Classification
C1	0.033	O
C2	0.160	X
C3	0.095	X
C4	0.145	X
C5	0.155	X
C6	0.040	O
C7	0.033	O
C8	0.036	O
C9	0.104	X
C10	0.047	O
C11	0.063	O
C12	0.039	O
C13	0.218	X
C14	0.257	X

Table S4. **Root mean square displacement of atoms during local structure relaxation to obtain the metastable configuration (C_6-SAM configurations).** C1, C2, …, C14 are cluster labels (see Figure S4). O and X indicate that the relaxed configurations were placed into the correct cluster and incorrect cluster, respectively, upon repeating the hierarchical clustering process, as shown in Figure S3.

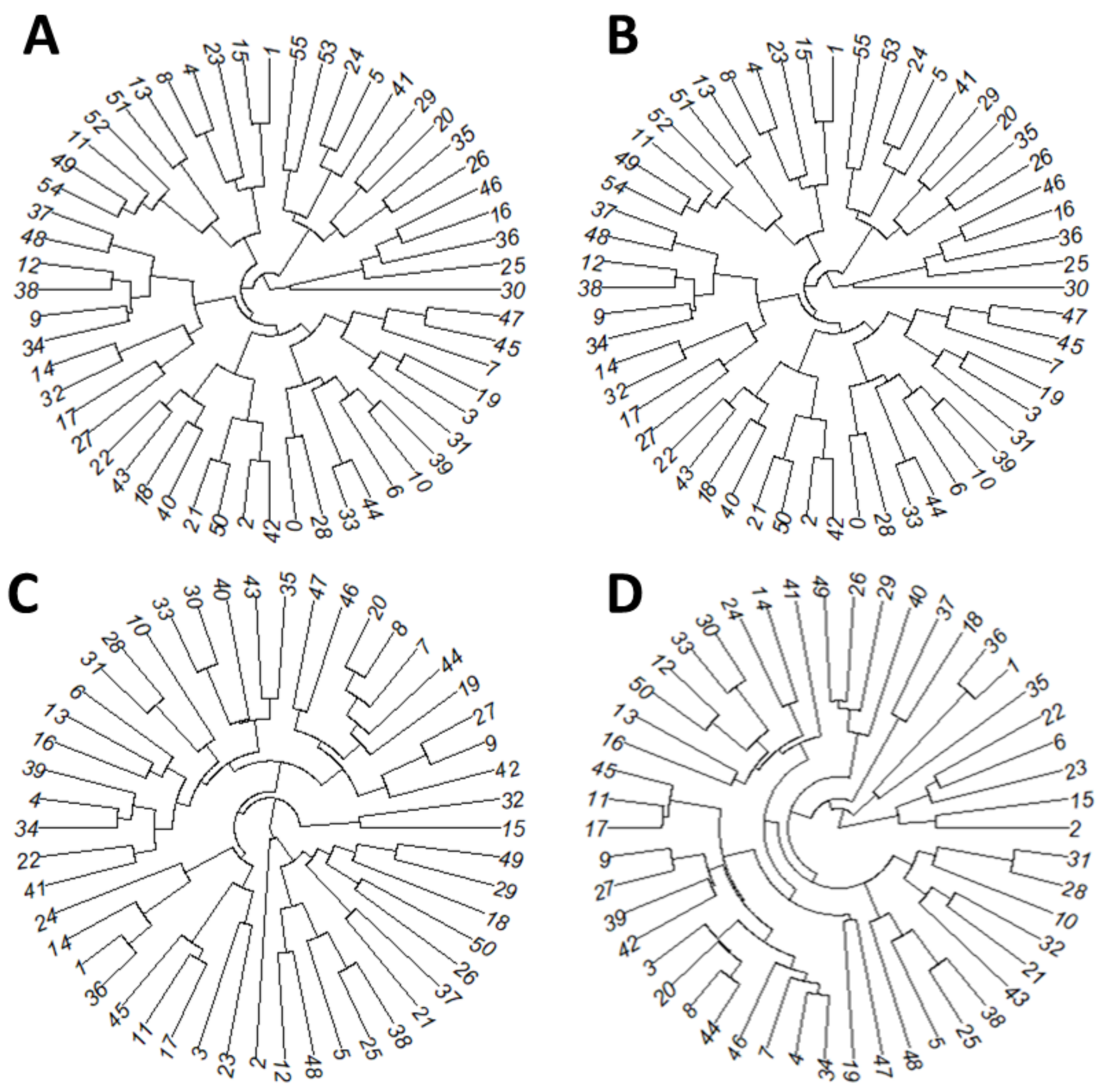

Figure S5. **Dendrograms computed with non-self-consistent potentials**. (A) Dendrogram from Figure 3A of the main paper, computed using self-consistent electrostatic potentials. (B) As for (A), but computed using non-self-consistent electrostatic potentials. (C) Dendrogram from Figure 5A of the main paper, computed using self-consistent potentials. (D) As for (C), but computed using non-self-consistent potentials. Note that we used t_0 = 1.03 fs and t_1 = 2.40 fs when computing (C) and (D).
